# Coupling phosphate-solubilizing bacteria (PSB) with inorganic phosphorus fertilizer improves mungbean (*Vigna radiata*) phosphorus acquisition, nitrogen fixation, and yield in alkaline-calcareous soil

**DOI:** 10.1016/j.heliyon.2022.e09081

**Published:** 2022-03-09

**Authors:** Hamid Khan, Waqas Ali Akbar, Zahir Shah, Hafeez Ur Rahim, Ali Taj, Juha.M. Alatalo

**Affiliations:** a Department of Soil and Environmental Sciences, The University of Agriculture, Peshawar, Pakistan; b Key Laboratory of Industrial Ecology and Environmental Engineering (Ministry of Education), School of Environmental Science and Technology, Dalian University of Technology, Dalian, 116024, China; c Environmental Science Center, Qatar University, PO Box 2713, Doha, Qatar

**Keywords:** Phosphate-solubilizing bacteria (PSB), Mineral phosphorus, Mungbean phosphorus acquisition, Nitrogen fixation, Yield, Alkaline-calcareous soils

## Abstract

To overcome the problems associated with soil phosphorus (P) insolubility, soil inoculation with phosphate-solubilizing bacteria (PSB) can be used. In a field experiment, we evaluated the efficacy of PSB in enhancing mungbean P acquisition, nitrogen (N) fixation, and morphological and yield traits in alkaline-calcareous soil when added together with P as single superphosphate (SSP) or rock phosphate (RP) at 45 or 90 kg P_2_O_5_ ha^−1^. Coupling PSB with mineral P fertilizers (SSP & RP) improved P use efficiency, mungbean P acquisition, N_2_ fixation, nodulation, NP uptake, and the morphological and yield-related traits of mungbeans compared with non-fertilized controls and plots received P from mineral sources alone. Soil PSB inoculation with mineral P also improved post-harvest soil fertility relative to pre-harvest by improving soil organic matter from 0.61% to 0.70%, lowering pH from 7.74 to 7.68, and improving soil total N from 0.04 to 0.09%, ABDTPA-extractable P from 2.07 to 3.44 mg kg^−1^, and potassium (K) concentrations from 100.27 to 129.45 mg kg^−1^. When combined with PSB, RP generally performed better than SSP. Moreover, there was a significant correlation between soil N and plant N, while the correlation between soil P and plant P was non-significant. The correlation between soil organic matter content and NP uptake by mungbeans was also non-significant. Therefore, adding P as RP at 45–90 kg ha^−1^, together with PSB inoculation, can be recommended for improving mungbean P acquisition, use efficiency, optimum N_2_ fixation, and yield in alkaline-calcareous soils.

## 1 Introduction

Phosphorus (P) is a major and essential macronutrient for food production and it plays a key role in different growth processes occurring in plants, such as root production, flowering, seed formation, photosynthesis, and maturation. The unavailability of soil P to plants due to binding to soil mineral particles and elements (e.g., calcium (Ca), magnesium (Mg), aluminum (Al), iron (Fe)) present in the soil causes severe crop yield losses (Lun et al., 2018; Roy et al., 2016). To overcome this problem, P fertilizers from different sources are applied at various levels to farm soils worldwide to meet plant P demand and recharge soil P reserves (Ramaekers et al., 2010). However, the problem with artificial fertilization is that only 25–30 % of the P applied is available to crops, while the remainder is converted into insoluble P fractions (Penn and Camberato, 2019). Therefore different management strategies, including the use of efficient targeted P fertilizers, organic amendments, plants with low critical P-requirements, and modified farming systems, have been devised to increase P use efficiency in soil systems (Du et al., 2022; Qaswar et al., 2021; Ahmed et al., 2021; Simpson et al., 2011). However, there are limitations on how well these management strategies can improve P use efficiency. As an alternative, the use of soil microbes, which can promote P solubility, use efficiency, and crop productivity, has been suggested (Elhaissoufi et al., 2021). Inoculation with beneficial microorganisms, as a form of biofertilizer, could then be used to replace the high inputs of chemical fertilizers in crop production (Billah et al., 2019; Kennedy et al., 2004).

In this regard, beneficial microbes such as phosphate-solubilizing bacteria (PSB), usually found in the rhizosphere of most plants, are attracting particular attention because of their reported advantages for alkaline calcareous soils (Elhaissoufi et al., 2021; Jilani et al., 2021). The soils in Pakistan are calcareous in nature and alkaline in reaction, which causes unavailability of P and N for plant uptake and growth promotion (Rahim et al., 2020). In such soils, the use of PSB can be beneficial, as these bacteria secrete phenolic compounds, protons (Ryan et al., 2001), and organic (Chen et al., 2006) and mineral acids (He and Zhu, 1998) into the soil, resulting in soil acidification (Jones, 1998) and subsequent P release from Ca_3_(PO_4_)_2_. The organic acids secreted by PSB also chelate cations such as Ca^2+^, Al^3+^, and Fe^3+^, and may increase bioavailable P (Adnan et al., 2020).

Phosphate-solubilizing bacteria may also improve P availability and crop growth by promoting biological nitrogen fixation (Chaiharn and Lumyong, 2011; Li et al., 2020b), through releasing growth promoters such as indoleacetic acid (Pathan et al., 2018), gibberellins, and cytokinins (Kucey et al., 1989). Additionally, PSB inoculation has been found to improve the yield and P nutrition of crops such as rice (Pal, 1998), maize (Afzal et al., 2005), and other cereals (Krishnaraj and Dahale, 2014). Thus, PSB can be an efficient, environmentally friendly and economically beneficial substitute for expensive P fertilizers. However, the potential of PSB in soils of a calcareous nature and with an alkaline reaction has not been well documented. The objectives of the present study were therefore to investigate the effect of PSB on nodulation, biological N_2_ fixation, and yield of mungbeans (*Vigna radiata*), to determine the appropriate P source and level, and to identify possible breakpoints and correlations between PSB and P sources and levels in nutrients (P, N) deficient alkaline-calcareous soils for growth and yield, N fixation, and P availability in mungbeans.

## 2 Materials and methods

### 2.1 Experimental set-up

**Figure 1:**
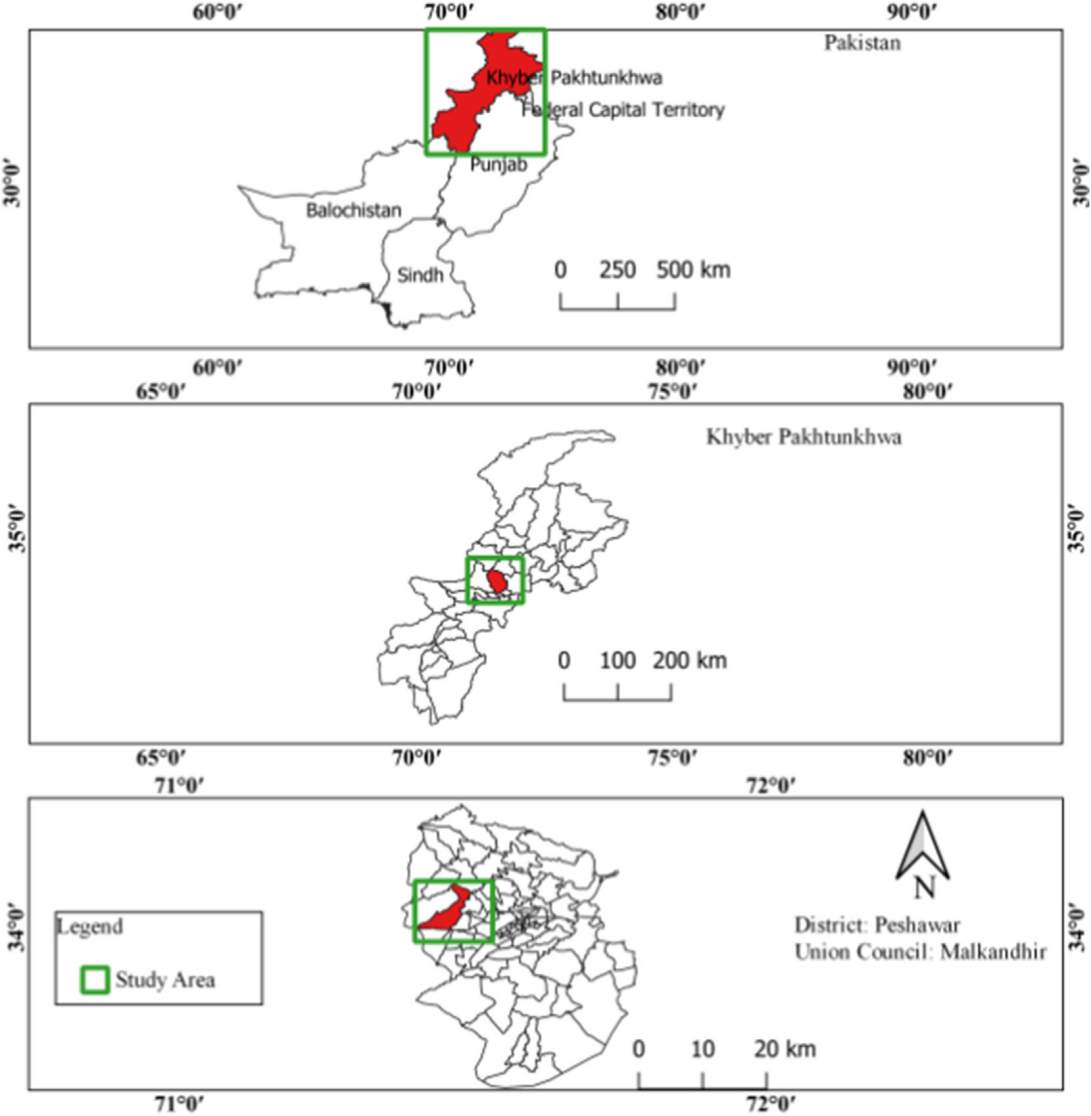
Geographical location of the study site (Agricultural Research Farm, The University of Agriculture Peshawar, Pakistan).

A field experiment during summer 2017 was conducted at the agricultural research farm at the University of Agriculture, Peshawar, Pakistan, located at 34.1^o’^ 21″N, 71^o^ 28′5″E (Figure 1) to assess the influence of sources and levels of P alone, or in combination with PSB, on P availability, use efficiency, N fixation, and yield of mungbean, which was used as a test crop. During the period of field experiment, the Peshawar city had a soil temperature of 9.27–33.01 °C, with an average temperature of 22.54 °C, while the air temperature were within the range of 9.52–36.6 °C, with an average temperature of 22.06 °C. Monthly mean relative humidity and rainfall were 50.44 % and 10.015mm, respectively (Fig. S1). Phosphorus was applied in the form of two different compounds, single superphosphate (SSP) and rock phosphate (RP), at a rate of 0 (control), 45, or 90 kg P_2_O_5_ ha^−1^, with or without PSB inoculation. The experiment was laid out in a randomized complete block design with three replicate plots per treatment, with each plot measuring 3 m × 3 m. The mungbean variety “Ramazan” was planted at 0.45 m spacing, accommodating a total of six rows per treatment plot. Phosphate fertilizer (SSP or RP) was applied before sowing and PSB inoculant in granular form was broadcast in plots just before the first irrigation. The required plant population was maintained manually by thinning when necessary. Standard agronomic practices and plant protection measures were used during crop growth and development, to keep the plots free of weeds, insect pests, and other diseases.

### 2.2 Treatments

The PSB product (ID: SOA (Ext) 1–70/2006) used in this study was obtained from the biofertilizer company Green Revolution (Pvt.) Ltd, Lahore, Pakistan, which imported the culture from Australia. The culture mainly comprised two P-solubilizing bacterial species (*Bacillus mageterium* and *Bacillus polymyxa*), together with other species. The characteristics of the product, based on information provided by the supplier, are shown in Table S1. Moreover, the detailed characteristics, population, and composition of the applied PSB in this research study chas been previously reported by Adnan et al. (2020). Details of the phosphate products and levels tested in the experiments are shown in Table S2, while the treatments applied to experimental plots are listed in Table 1.

**Table 1:** Treatment combinations applied in plots T1-T10. SSP = single superphosphate, RP = rock phosphate, PSB = phosphate-solubilizing bacteria (5 kg ha^−1^).

Treatment	Factor A:	Factor B	Factor C
	P source	P level (kg ha^−1^)	PSB
T1	Control	0	No PSB
T2	SSP	0	Only PSB
T3	SSP	45	Without PSB
T4	SSP	90	Without PSB
T5	SSP	45	With PSB
T6	SSP	90	With PSB
T7	RP	45	Without PSB
T8	RP	90	Without PSB
T9	RP	45	With PSB
T10	RP	90	With PSB

### 2.3 Soil sampling, processing, and analysis

Soil samples at a 0–30 cm depth from the experimental site were collected twice using a zigzag approach, before the start of the experiment (pre-harvest sampling) and at the end of the experiment (post-harvest sampling). The pre-harvest sampling was representative of the experimental site soil, while the post-harvest soil sampling was representative of each experimental unit that received different treatments. The pre and post soil samples were air-dried, milled to pass through a 2-mm sieve, and analyzed for the selected parameters. The soil pH was measured in a soil water suspension of 1:5 as prescribed by McLean (1983), soil organic matter by Nelson and Sommers (1983), soil and plant total nitrogen by Bremner (1996), AB-DTPA extractable P and K in soil by Soltanpour and Schwab (1977), and total P and K in plants were measured by Kuo (1996). The pre-harvest analysis of the soil at the experimental site revealed that it is calcareous in nature, alkaline in reaction, and deficient in phosphorous and nitrogen (Table 2).

**Table 2:** Physical and chemical properties of the soil at the experimental site before the start of the experiment.

Property	Value
Silt content	54.7%
Sand content	23.5%
Clay content	21.8%
Textural class	Silty clay loam
pH	7.74
Electrical conductivity	0.17 d S m^−1^
Bulk density	1.34 g cm^−3^
Organic matter content	0.61%
AB-DTPA-extractable P	2.07 mg kg^−1^
AB-DTPA extractable K	100.27 mg kg^−1^
Total N	0.04%
Lime content	15%

### 2.4 Morphological and yield related traits

Data on morphological and yield-related traits, such as nodules per plant, fresh and dry weight of nodules (g plant^−1^), pods per plant, seeds per plant, biological yield (kg ha^−1^), grain yield (kg ha^−1^) 1000-grain weight (g), and N fixation were collected and analyzed using standard procedures (Majeed et al., 2020). The detailed procedures are elaborated here.

To estimate nodules per plant, three randomly selected plants were carefully uprooted with the help of a spade at the pod's development stage from each sub-treatment plot. Plant roots were washed with water to remove soil. After complete removal of soil, nodules were counted on the roots of each plant. Nodules of three plants were summed for each treatment plot and an average was taken. To estimate fresh and dry weight of nodules (g plant^−1^), nodules were detached from three plants from each treatment plot and weighed first for fresh weight and then dried in an oven at 105 °C for 24 h and the dry weight recorded. To estimate pods per plant, ten plants were randomly selected in each treatment plot, and pods were counted on each plant. The pods were summed, and the average was calculated per plant. To estimate number of seeds per plant, from each treatment plot, ten pods were randomly selected, and seeds in each pod were counted. Seeds of all ten pods were summed, and the average was taken per pod data. To estimate biological yield, an area of 1 m^2^ was harvested in each treatment plot and weighed for fresh biomass. The harvested biomass was dried in shade for 4 days, and reweighed for dry biomass biological yield, and converted into kg ha^−1^. To estimate grain yield, an area of 1 m^2^ was harvested in each treatment plot and threshed. After cleaning, grain weighed was recorded. The yield was then converted into kg ha^−1^. For the thousand grain weight, a thousand grains were collected and weighed.

Harvest index was determined using the following equation.

(1) $$\text{Harvest ​index}=\frac{\text{Economic ​yield}\left(\text{Kg ​}{\text{ha}}^{-1}\right)}{\text{Total ​dry ​biomass ​}\left(\text{Kg ​}{\text{ha}}^{-1}\right)}\times 100$$

Total N uptake (kg ha^−1^) in the crop was determined using the values of dry plant biomass (kg ha^−1^) and its N concentration as follows:

(2) $$Total\;N\;uptake\;\left(kg\;ha^{-1}\right)=\%\;N\;concentration\times dry\;biomass\;\left(kg〖\;ha〗ˆ(-1)\right)\;÷100$$

N_2_ fixation was determined by subtracting N uptake in reference plant biomass from the N uptake in mungbean plant biomass using the following formula:

(3) $$N^{2}fixed\;\left(kg\;ha^{-1}\right)=N\;uptake\;\left(kg\;ha^{-1}\right)\;in\;mungbean-N\;uptake\;in\;reference\;\left(kg\;ha^{-1}\right)$$

### 2.5 Statistical analysis

The data obtained on various parameters was analyzed statistically using analysis of variance (ANOVA) techniques. Significant differences in means of treatments at a 5% (P < 0.05) level of probability were separated using the least significant difference (LSD) test by statistix 8.1.

## 3 Results and discussion

### 3.1 Effect of PSB and P fertilizers on number of nodules per plant, fresh & dry weight of nodules, and N_2_ fixation

The Number of nodules recorded per plant, fresh weight and dry weight of nodules, and N_2_ fixation in the treatments with inorganic P sources (SSP, RP) at 45 and 90 kg ha^−1^, without and with PSB addition, are shown in Table 3. Application of P fertilizer (P and SSP) significantly increased the number of nodules per plant from 22 (control) to 29 (SSP) or 30 (RP). The number of nodules per plant also significantly increased, from 28 to 30, with P level increasing from 45 to 90 kg ha^−1^. The application of PSB alone significantly increased the number of nodules per plant (from 27 to 31) compared with the treatments without PSB.

**Table 3:** Effect of phosphorus (P) source, P level, and addition of phosphate-solubilizing bacteria (PSB) on nodulation and nitrogen fixation in mungbean.

Treatment	Number of nodules plant^−1^	Fresh weight of nodules plant^−1^ (g)	Dry weight of nodules plant^−1^(g)	Amount of N_2_ fixed (kg ha^−1^)
Control	22	1.27	0.37	35.86
All treatments	29	1.40	0.52	105.64
Significance level	∗∗	∗	∗∗	∗∗
P source (PS):				
Single superphosphate	27b†	1.30b	0.52	94.34
Rock phosphate	30a	1.46a	0.49	129.95
Significance level	∗∗	∗∗	ns	∗∗
P level (PL):				
45 kg ha^−1^	28b	1.34	0.48	107.16
90 kg ha^−1^	30a	1.42	0.53	117.13
Significance level	∗	ns	ns	∗∗
PSB addition:				
Without PSB	27b	1.30b	0.47b	93.11
With PSB	31a	1.46a	0.54a	131.18
Significance level	∗∗	∗∗	∗∗	∗∗
Interactions:	significance level			

PS x PL	ns	ns	ns	ns
PS x PSB	ns	ns	ns	∗∗
PL x PSB	ns	ns	ns	ns
PS x PL x PSB	ns	ns	ns	∗∗

† Different letters after values indicate statistically significant difference (LSD test) at ∗P < 0.05; ∗∗P < 0.01, ns = non-significant.

The fresh weight of nodules showed significant differences between the P fertilizer treatments (‘all treatments’ in Table 3) and the control. In addition, the RP treatment produced a significantly greater fresh weight of nodules than the SSP treatment. However, no significant differences in fresh weight of nodules in mungbeans were observed between the two P levels tested (45 and 90 kg P_2_O_5_ ha^−1^), or between the treatments with and without PSB.

A rather similar trend for dry weight of nodules as observed for the fresh weight of nodules was found for the different treatments, with the dry weight of nodules being significantly greater in the P fertilizer treatments than in the control and no significant differences among the P sources and levels. However, the application of PSB significantly affected the dry weight of nodules, with higher dry weight observed with the application of PSB compared with no PSB treatment. Nitrogen (N_2_) fixation by mungbean was found to be significantly greater in treatments received RP than in those received SSP. Application of PSB with both P fertilizers substantially increased the amount of N_2_ fixed in mungbean.

**Figure 2:**
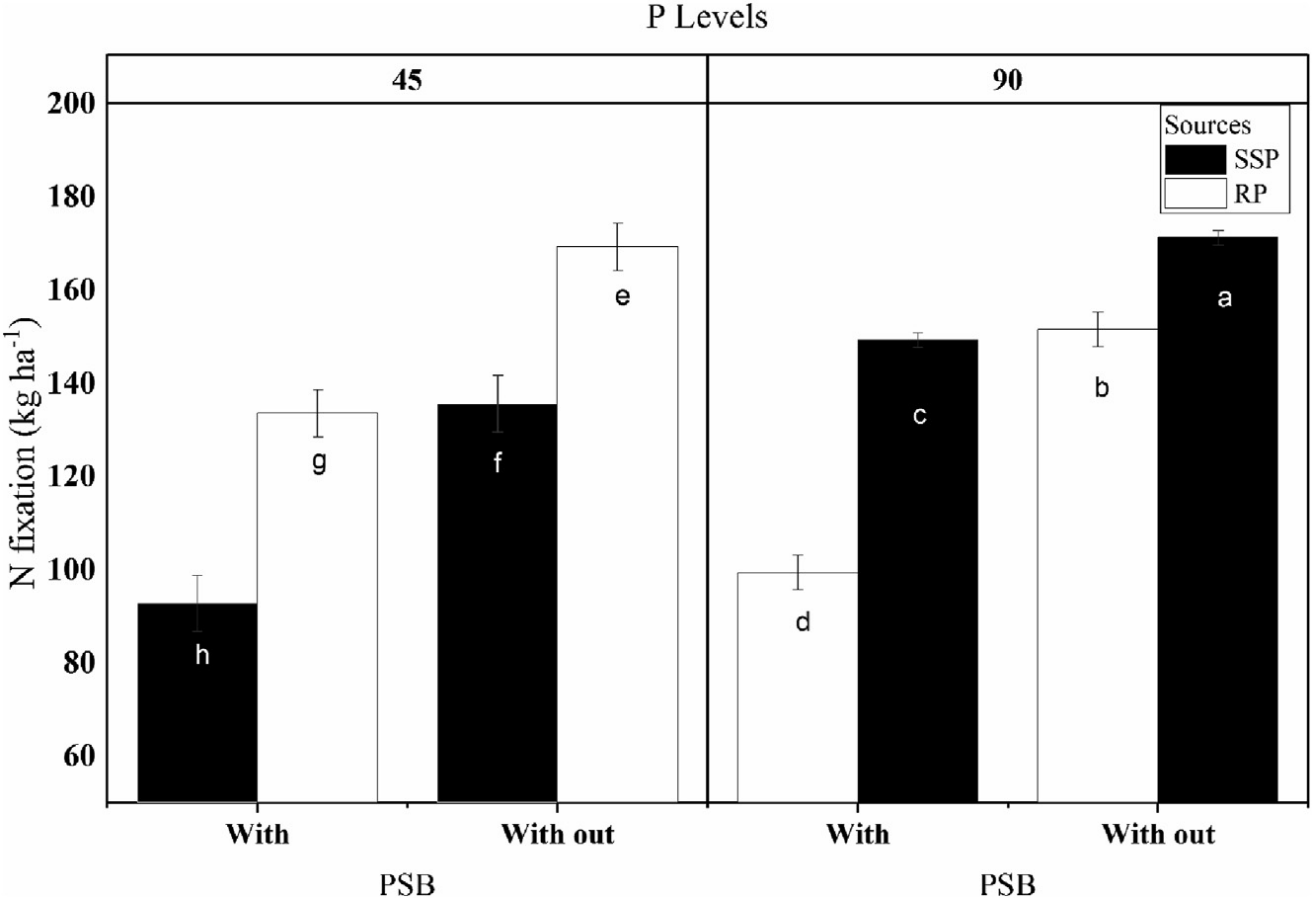
Interactive effect of PSB, P source (SSP) or (RP), and P levels (45 or 90 kg ha^−1^) on nitrogen fixation in mungbean. Different letters over the bars represent the significance (P < 0.05) differences among treatments according to LSD test. Replication number (n = 3).

Analysis of the results revealed significant interactions between P source × PSB and P source × P level × PSB for N_2_ fixation in mungbeans (Table 3, Figure 2). These results are in line with findings in a previous study where inoculation of mungbeans with *Bacillus megaterium* significantly increased the number of nodules compared with the control (Korir et al., 2017). The significant difference seen in the number of nodules with and without PSB partly confirms previous findings that PSB has the potential to significantly increase nodulation, in terms of nodule number and nodule fresh and dry weight, in both mungbeans and maize (Ahmad et al., 2019). It has also been shown that N fixation in soybeans can be improved by P application (Yong et al., 2018). This increase may be attributable to more numerous and healthier nodules due to more P being present in the rhizosphere, promoting root development (Li et al., 2020b).

### 3.2 Effect of PSB and P fertilizers on N & P concentration in plants & uptake by mungbeans

Plant P and N concentrations and their uptake by mungbean crops under different P sources and levels, in the presence and absence of PSB, are shown in Table 4. Significant differences in plant P concentration were observed between all treatments. The P concentration in mungbean plants was slightly higher for SSP than RP (SSP˃RP), but the difference was statistically non-significant (P < 0.05). The P concentration in plants increased significantly with the increasing level of P application. Application of PSB also increased the P concentration in mungbean plants, from 0.26 % in the absence of PSB to 0.34 % when PSB was present. The interaction between P source × PSB was found to be significant, but other interactions were non-significant (P < 0.05). Total P uptake by mungbeans followed an opposing trend for the two forms of P fertilizer (RP˃SSP) compared with that seen for plant P concentration (SSP˃RP). Moreover, P uptake by the mungbean crop increased significantly with increasing P level (Table 4). Similarly, P uptake increased notably with the application of PSB (30.20 kg ha^−1^) compared with no PSB treatment (22.15 kg ha^−1^).

**Table 4:** Effect of phosphorus (P) source, P level, and addition of phosphate-solubilizing bacteria (PSB) on P and nitrogen (N) uptake by mungbean.

Treatment	P concentration in plant (%)	N concentration in plant (%)	Total uptake P (kg ha^−1^)	Total uptake N (kg ha^−1^)
Control	0.18	0.92	11.49	58.01
All treatments	0.29	1.19	25.43	103.35
Significance level	∗∗	∗∗	∗∗	∗∗
P source (PS):				
Single superphosphate	0.31	1.15b†	24.00	96.52
Rock phosphate	0.29	1.26a	28.36	115.81
Significance level	Ns	∗	∗∗	∗∗
P level (PL):				
45 kg ha^−1^	0.28b	1.15b	24.64	100.14
90 kg ha^−1^	0.31a	1.26a	27.71	112.19
Significance level	∗	∗	∗∗	∗∗
PSB addition:				
Without PSB	0.26b	1.11	22.15	95.99
With PSB	0.34a	1.30	30.20	116.33
Significance level	∗∗	∗∗	∗∗	∗∗
Interactions:	significance level			

PS x PL	ns	ns	Ns	ns
PS x PSB	∗∗	ns	∗∗	ns
PL x PSB	ns	ns	Ns	ns
PS x PL x PSB	ns	ns	Ns	ns

† Different letters after values indicate statistically significant difference (LSD test) at ∗P < 0.05; ∗∗P < 0.01, ns = non-significant.

The N concentration in mungbean plants was significantly higher in RP-treated plots than in SS-treated plots (RP˃SSP). It was observed that increasing the level of P application had a significant effect on N concentration in plants. Moreover, the N concentration in mungbeans was higher in the presence of PSB than in the absence of PSB. Total N uptake by mungbeans followed a similar trend (RP˃SSP) as that seen for N concentration in mungbean plants (RP˃SSP) and N uptake by the mungbean crop increased significantly with increasing N level. Additionally, N uptake increased notably with the application of PSB compared with no PSB treatment. A previous study found that nitrogen content and uptake by mungbeans were significantly influenced by *Rhizobium* inoculant and P fertilization, with N content in shoots at harvest varying from 3.2 % to 4.2 % (Rahman et al., 2008). Later studies found that PSB inoculation significantly increased N and P uptake in mungbeans (Rani et al., 2016), and that increasing levels of P fertilizer (from 20 to 40 kg ha^−1^) increased plant N content in mungbeans (Sipai et al., 2015). Recently, it was reported that a consortium of endophytes and PSB increased P concentration and use efficiency in wheat cultivars grown on P-deficient soils (Emami et al., 2020). Similarly, Estrada-Bonilla et al. (2021) reported that the inoculation of PSB enhances the availability and use efficiency of P in the sugarcane-soil system. Bargaz et al. (2021) recently reviewed how the co-application of PSB and P in soil could enhance the efficient utilization of P for sustainable cropping systems and ensure the judicious use of mineral nutrients.

### 3.3 Effect of PSB & P fertilizer on mungbean biomass (fresh & dry) and yield parameters

The results obtained for fresh biomass, dry biomass, grain yield, pods per plant, seeds per pod, and 1000-grain weight are shown in Table 5. Fresh biomass of mungbeans was significantly greater in all P fertilizer and PSB treatments compared with the control, but there was greater variation in fresh plant biomass in the P fertilizer treatments, with RP producing the most biomass. Moreover, a substantial increase in fresh plant biomass, from 13508 to 15025 kg ha^−1^, was observed with P fertilizer level increasing from 40 to 90 kg ha^−1^. Similarly, a substantial increase in fresh biomass, from 12683 to 15880 kg ha^−1^, was obtained with PSB compared with no PSB. The interaction between P source ×PSB was significant, but other interactions were non-significant (P < 0.05).

**Table 5:** Effect of phosphorus (P) source, P level, and addition of phosphate-solubilizing bacteria (PSB) on plant biomass (kg ha^−1^) of mungbean.

Treatment	Fresh biomass	Dry biomass	Grain yield	No. of pods per plant	No. of seeds per pod	1000-grain weight (g)
Control	8700.00	6333.33	1137	21.67	11.00	43.31
All treatments	13970.37	8625.93	1641	25.67	12.89	48.28
Significance level	∗∗	∗∗	∗∗	∗∗	∗∗	ns
P source (PS):						
Single superphosphate	13016.67b†	8358.33b	1626	24.42	12.58b	49.87
Rock phosphate	15516.67a	9191.67a	1587	26.08	13.00a	49.67
Significance level	∗∗	∗∗	ns	ns	∗	ns
P level (PL)						
45 kg ha^−1^	13508.33b	8666.67b	1614	24.67	12.83	46.75b
90 kg ha^−1^	15025.00a	8883.33a	1600	25.83	12.75	52.79a
Significance level	∗∗	∗	ns	ns	ns	∗
PSB addition:						
Without PSB	12683.33b	8650.00b	1524b	23.50b	12.58b	43.45b
With PSB	15850.00a	8900.00a	1690a	27.00a	13.00a	56.09a
Significance level	∗∗	∗	∗∗	∗∗	∗	∗∗
Interactions:	significance level					

PS x PL	ns	ns	ns	ns	ns	ns
PS x PSB	∗∗	ns	ns	ns	ns	∗∗
PL x PSB	ns	ns	ns	ns	ns	ns
PS x PL x PSB	ns	ns	∗	ns	ns	∗∗

† Different letters after values indicate statistically significant difference at ∗P < 0.05; ∗∗P < 0.01, ns = non-significant.

Dry biomass followed the same trend as fresh plant biomass, i.e., it was greater for RP than SSP and increased with increasing levels of P application, from 6333 kg ha^−1^ in the control to 8667 kg ha^−1^ at 45 P kg ha^−1^ and 8883.33 kg ha^−1^ at 90 P kg ha^−1^. Moreover, dry plant biomass was significantly greater in the presence than in the absence of PSB (8900 compared with 8650 kg ha^−1^). However, all the interactions between treatments were statistically non-significant (P < 0.05).

Higher grain yield was obtained in the P fertilizer and PSB treatments in comparison with the control, but grain yield was not significantly affected by the level or source of P. The PSB treatment produced a considerably greater grain yield of mungbeans (1690 kg ha^−1^) compared with the non-PSB treatment (1524 kg ha^−1^). However, all the interactions except P source × P level × PSB were statistically non-significant.

Number of pods per plant was considerably greater in the P and PSB treatments compared with the control. It was not notably affected by the level or source of P fertilizer, but it was significantly greater in the presence of PSB (27) compared with no PSB (23.5). However, all interactions were non-significant. The RP and PSB treatments produced more seeds per pod than SSP and all other combined treatments, with the highest number of seeds per pod produced in the treatments that received PSB with P application. In terms of 1000-grain weight of mungbeans, there were no significant differences between the treatments, although a slight increase was observed between the control and treated plots. The interactions between the treatments were also found to be non-significant. Similarly, a previous study found that inoculation of seed with PSB significantly increased the number of pods per plant, number of seeds per pod and yield of mungbeans, with an increase of 3.88 % in seed and 3.99 % in stover yield of mungbeans with PSB inoculation compared with the uninoculated control (Rani et al., 2016). Another study reported remarkable increases in mungbean growth, yield, and N fixation with the application of P fertilizer and rhizobacteria strains (Yadegari et al., 2010). The improvement in morphological and yield-related traits of mungbean in plots with the addition of PSB, compared with plots that received P from mineral sources alone, can be attributed to the ability of PSB to release bound P from both organic and inorganic sources to plants under the action of functionally diverse groups and growth regulators secreted by PSB (Khan et al., 2013).

### 3.4 Effect of PSB and P fertilizer on post-harvest soil properties

**Figure 3:**
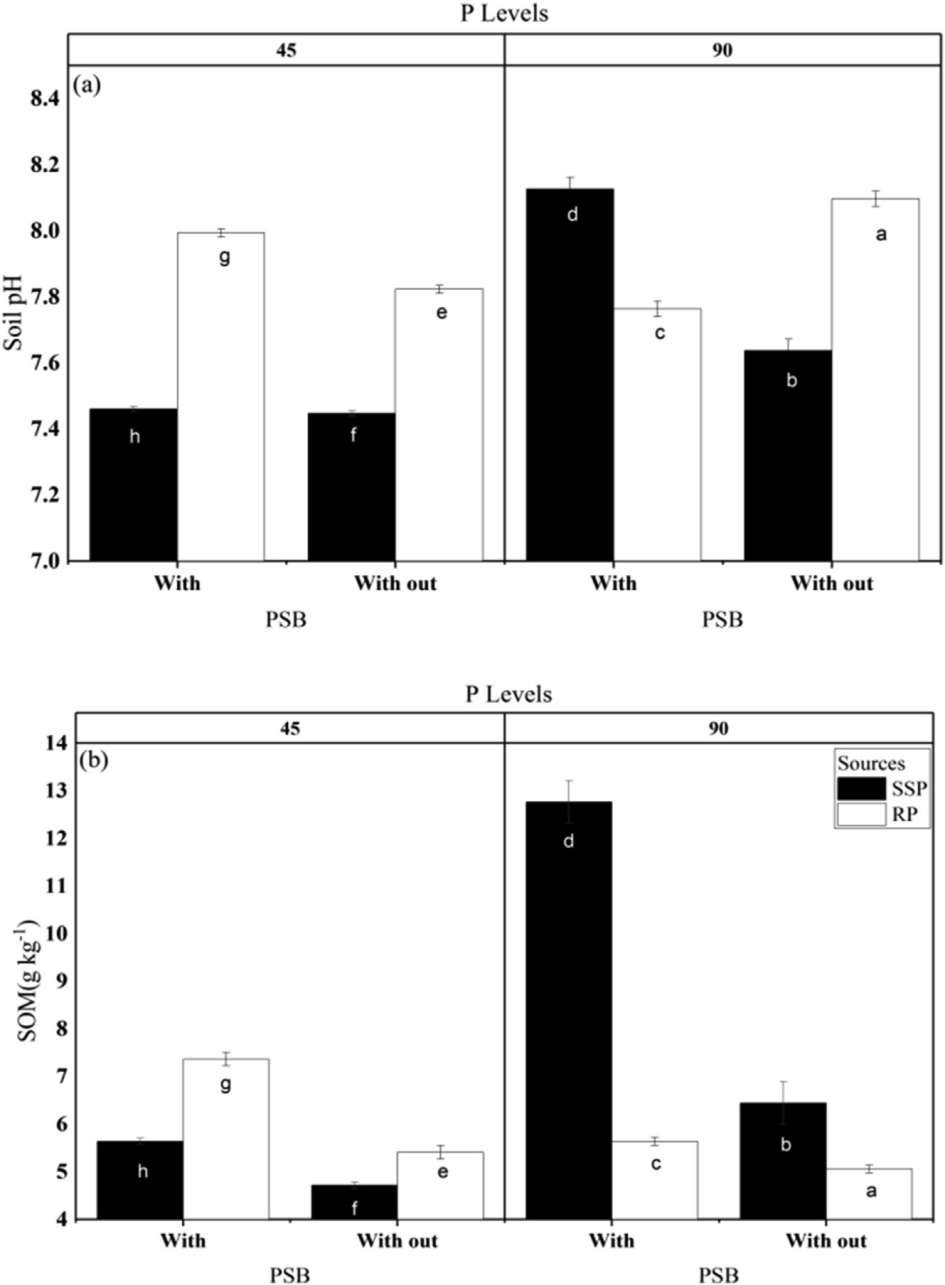
Interactive influence of PSB, P source (SSP) or (RP), and P level (45 or 90 kg ha^−1^) on (a) soil pH and (b) soil organic matter (OM) content. Different letters over the bars represent the significance (P < 0.05) differences among treatments according to LSD test. Replication number (n = 3).

The values obtained for soil pH, organic matter content, AB-DTPA-extractable P, total N, and AB-DTPA-extractable K after mungbean harvest are shown in Table 6. No significant differences in soil pH were observed between treatments and the control, but soil pH was significantly greater in the RP-amended soil and also increased considerably with increasing level of P applied. No significant differences in soil pH were observed between P treatments in the presence and absence of PSB, but soil pH was considerably reduced with PSB alone. The interactions P source × P level, P source × PSB and P source × P level × PSB were significant for soil pH (Figure 3). A substantial decrease in soil pH with the application of PSB in combination with RP has been reported previously (Chen et al., 2006).

**Table 6:** Effect of phosphorus (P) source, P level, and addition of phosphate-solubilizing bacteria (PSB) on soil pH and soil content of organic matter (OM) content, P, nitrogen (N) and potassium (K) after harvest of mungbean.

Treatments	Soil pH	Soil organic matter (%)	Soil P (mg kg^−1^)	Soil N (%)	Soil K (mg kg^−1^)
Control	7.74	0.61	2.07	0.04	100.27
All treatments	7.68	0.70	3.44	0.09	129.45
Significance level	ns	Ns	∗∗	∗∗	∗∗
P source (PS):					
Single super phosphate	7.67b†	0.74a	3.66a	0.10	121.40b
Rock phosphate	7.92a	0.59b	3.09b	0.09	143.56a
Significance level	∗∗	∗∗	∗∗	ns	∗∗
P level (PL):					
45 kg ha^−1^	7.68b	0.58b	2.80b	0.09b	129.58b
90 kg ha^−1^	7.91a	0.75a	3.94a	0.11a	135.37a
Significance level	∗∗	∗∗	∗∗	∗	∗∗
PSB addition:					
Without PSB	7.84	0.78a	3.07b	0.08b	128.01b
With PSB	7.75	0.54b	3.67a	0.12a	136.95a
Significance level	ns	∗∗	∗∗	∗∗	∗∗
Interactions:	significance level				

PS x PL	∗∗	∗∗	∗∗	ns	ns
PS x PSB	∗	∗∗	∗∗	ns	∗∗
PL x PSB	ns	∗∗	∗∗	ns	ns
PS x PL x PSB	∗∗	∗∗	∗∗	ns	ns

† Different letters after values indicate statistically significant difference at ∗P < 0.05; ∗∗P < 0.01, ns = non-significant.

Organic matter content was slightly higher in amended soil compared with the control, but the differences were statistically non-significant (P < 0.05). In the plots with P fertilization, SSP produced more organic matter than RP. Soil organic matter was also significantly greater in the presence of PAB than in the treatments without PSB. All interactions (P source × P level, P level × PSB, P source × PSB, and P source × P level × PSB) were significant for accumulation of organic matter in the soil (Figure 3). These results are in agreement with the findings that application of RP with PSB can substantially increase soil organic matter (Ul Hassan and Bano, 2015).

**Figure 4:**
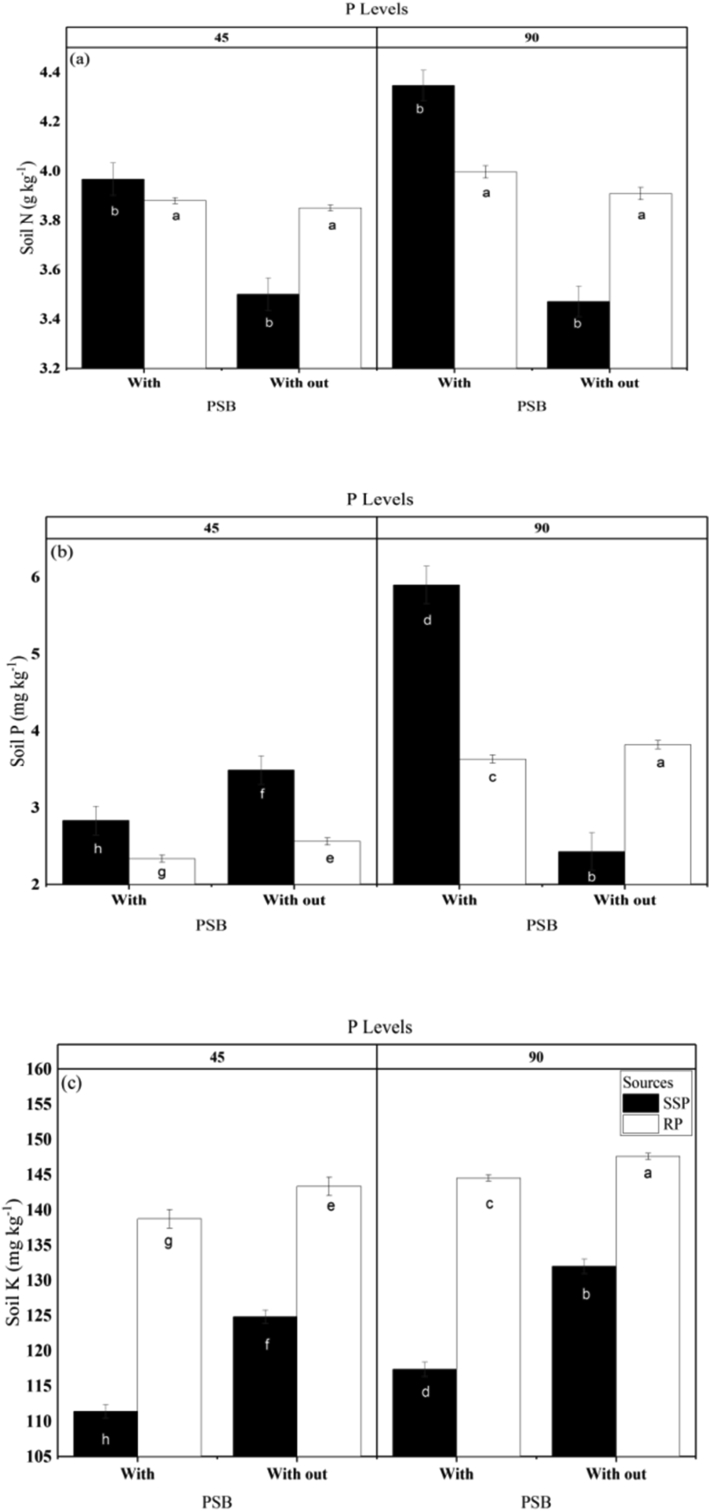
Interactive influence of PSB, P source (SSP) or (RP), and P level (45 or 90 kg ha^−1^) on soil (a) (N), (b) P, and (c) K. Different letters over the bars represent the significance (P < 0.05) differences among treatments according to LSD test. Replication number (n = 3).

Extractable P content in soil was significantly greater in the SSP treatment than in RP-treated soil and increased markedly with increasing levels of P application. It was also significantly greater in the presence than in the absence of PSB, and was enhanced in the PSB-no fertilizer treatment compared with other non-PSB treatments. The interactions P source × P level, P source × PSB and P source × P level × PSB were significant for soil extractable phosphorus (Figure 4). Similarly a previous study found that combined P fertilizer and PSB application resulted in a remarkable improvement in N and P content in soil, with 40 kg P_2_O_5_ + PSB giving the best results (Naik et al., 2013). Estrada-Bonilla et al. (2021) found that the co-application of PSB with compost increased the P content in soil and consequently their uptake in sugarcane shoots.

Our results showed that soil N was significantly higher in amended soil in comparison with the control and was slightly higher in the RP plots than in the SSP plots, although the differences were statistically non-significant (P < 0.05). The increasing level of P fertilizer had a significant increasing effect on soil N, as did the presence of PSB compared with no PSB. However, all interactions except P source × PSB were non-significant (Figure 4). An earlier study also found that increasing levels of P fertilizer resulted in a greater build-up of available N and P content in the soil after the harvest of a mungbean crop (Sipai et al., 2015). Estrada-Bonilla et al. (2021) found that the co-application of PSB with compost increased the N content in soil and consequently their uptake in sugarcane shoots.

Soil K content was significantly higher in soils that received RP than in SSP plots and increased with increasing levels of P fertilizer. Moreover, the application of PSB considerably increased the concentration of K in soil. However, all interactions except P source x PSB were non-significant (Figure 4). It has been shown that the concentration of K in the soil is increased by the regulation of organic acid metabolism and H^+^ secretion by PSB (Li et al., 2020a). Our results are in agreement with findings that the application of P can increase the K content in soil (Sharma et al., 2011). Estrada-Bonilla et al. (2021) found that the co-application of PSB with compost increased the K content in soil and consequently their uptake in sugarcane shoots.

### 3.5 Correlation between soil NP, soil organic matter, plant NP and their uptake

**Figure 5:**
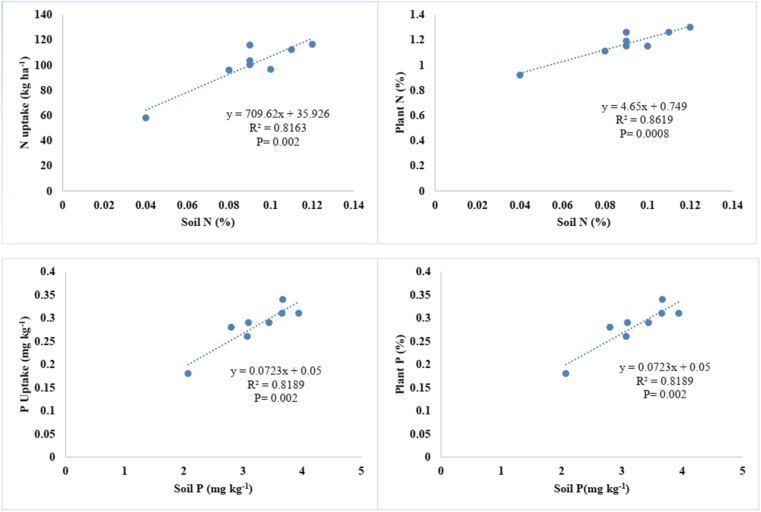
Correlations of soil nitrogen (N) and phosphorus (P) content with plant N and P content, and plant N and P uptake.

**Figure 6:**
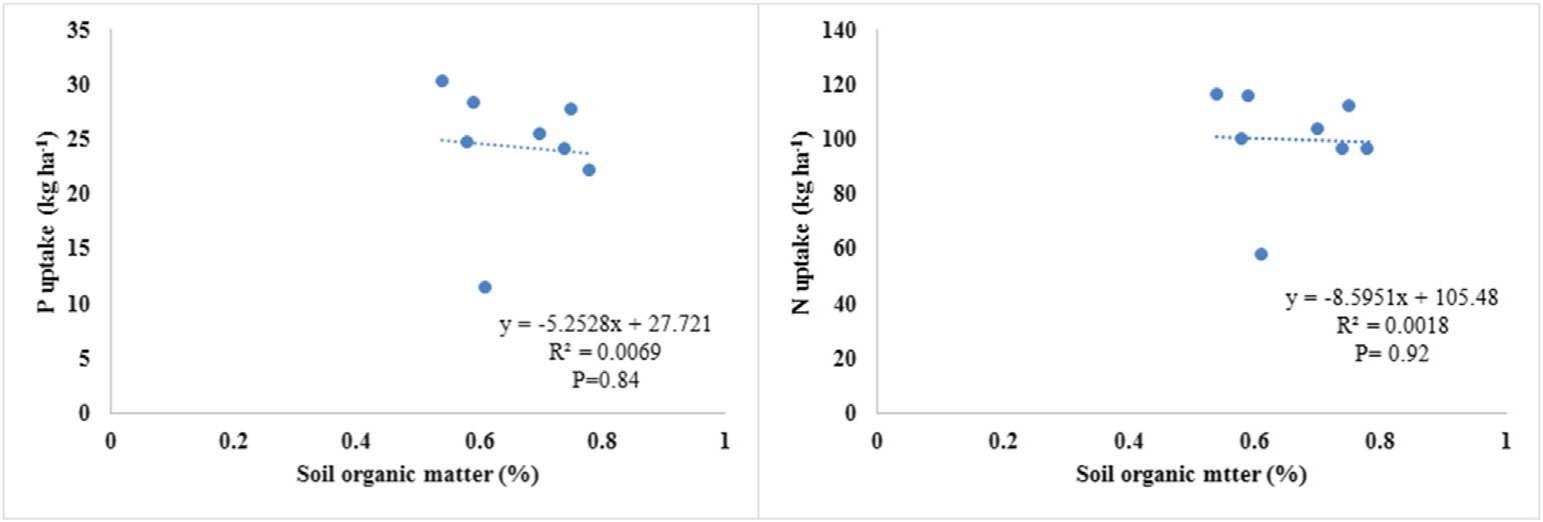
Relationships between soil organic matter (SOM) content and P uptake and N uptake by mungbean plants.

The relationships between soil N and P content, plant N and P content, and N and P uptake by mungbean plants are presented in Figure 5. As can be seen, the significant increase in soil N brought about by the treatments ultimately enhanced N uptake, leading to increased N content in the plants. The relationship of soil P with plant P and P uptake by mungbean crops was found to be non-significant. Yu et al. (2012) reported that PSB in combination with nitrogen fixing bacteria increased the solubilisation of RP in soil, and consequently enhanced the uptake of NP in soil. However, our results regarding the association between soil P and plant P were found to contrast with previous studies. In view of this, Sundara et al. (2002) reported that PSB application in conjunction with RP could increase the plant available status in soil. No significant correlation was found between soil organic matter, N uptake, and P uptake by mungbean plants (Figure 6). Our results are in contrast with Hussain et al. (2021), who reported that the relationship between soil organic matter, N and P uptake in PSB inoculated soil was strong, meaning that PSB in soil regulated the mineralization of organic matter, subsequently improving NP uptake.

## 4 Conclusions

Coupling PSB with mineral P fertilizers at different levels enhanced mungbean P acquisition, utilization efficiency, biological N2 fixation, root nodulation, NP uptake, and morphological and yield-related traits in comparison with control and mineral P application alone as SSP or RP. When combined with PSB, RP performed better than SSP. Our results confirmed that PSB in combination with P fertilizers performs well in alkaline-calcareous soils. Thus, it is suggested that PSB and P fertilizers, and especially RP could be applied in alkaline-calcareous soils. Further study on different PSB and P fertilizers levels, combinations, and P sources (organic/inorganic) in the soil-legumes-cover-crop and cereals crop system are suggested to get more in-depth insights.

## Declarations

### Author contribution statement

Hamid Khan; Ali Taj: Performed the experiments; Analyzed and interpreted the data; Contributed reagents, materials, analysis tools or data.

Waqas Ali Akbar: Analyzed and interpreted the data; Contributed reagents, materials, analysis tools or data.

Zahir Shah: Analyzed and interpreted the data; Contributed reagents, materials, analysis tools or data.

Hafeez Ur Rahim: Conceived and designed the experiments; Analyzed and interpreted the data; Contributed reagents, materials, analysis tools or data; Wrote the paper.

Juha. M. Alatalo: Conceived and designed the experiments; Analyzed and interpreted the data; Wrote the paper.

### Funding statement

J.M.A. was supported by 10.13039/501100004251Qatar Petroleum (QUEX-CAS-QP-RD-18/19).

### Data availability statement

Data will be made available on request.

### Declaration of interests statement

The authors declare no conflict of interest.

### Additional information

No additional information is available for this paper.

## Appendix A Supplementary data

Supplementary materialsRevised Supplementary materialsRevised
