# Supplementary material for: Coupling phosphate-solubilizing bacteria (PSB) with inorganic phosphorus fertilizer improves mungbean (Vigna radiata) phosphorus acquisition, nitrogen fixation, and yield in alkaline-calcareous soil
Source: Heliyon. 2022 Mar 9;8(3):e09081. doi: 10.1016/j.heliyon.2022.e09081 (PMC8919226; doi:10.1016/j.heliyon.2022.e09081)
Supplement: Supplementary materialsRevised [file mmc1.docx]

Coupling phosphate-solubilizing bacteria (PSB) with inorganic phosphorus fertilizer improves mungbean (*Vigna radiata*) phosphorus acquisition, nitrogen fixation, and yield in alkaline-calcareous soil

Hamid Khan^1^, Waqas Ali Akbar^1^, Zahir Shah^1^, Hafeez Ur Rahim^2,*^, Ali Taj^1^, Juha. M. Alatalo^3,*^

^1^Department of Soil and Environmental Sciences, The University of Agriculture, Peshawar, Pakistan.

^2^Key Laboratory of Industrial Ecology and Environmental Engineering (Ministry of Education), School of Environmental Science and Technology, Dalian University of Technology, Dalian 116024, China

^3^Environmental Science Center, Qatar University, PO Box 2713, Doha, Qatar

*Corresponding author’s email:

[hafeez.kalpani@aup.edu.pk](mailto:hafeez.kalpani@aup.edu.pk)

[jalatalo@qu.edu.qa](mailto:jalatalo@qu.edu.qa)

**

**

**Fig. S1. Atmospheric (agro-met) measurements during the field experiment.**

**Table S1.** Characteristics of the phosphate-solubilizing bacteria (PSB) product (ID: SOA (Ext) 1-70/2006) used in the experiment

| **PSB Characteristics** | **Unit** | **Magnitude** |
| --- | --- | --- |
| Population | CFU gm^-1^ | 1.5 x 10^7^ |
| Auxin | Mg ML^-1^ | 4.7 ± 0.53 |
| IAA | µg m L^-1^ | 7.5 ± 0.71 |
| P solubilisation | Diameter of halo in mm | 7.0 ± 0.42 |
| Siderophores | Diameter of halo in mm | 6.0 ± 0.66 |
| Material type | --- | Organic |
| Total organic acid | g L^-1^ | 11 ± 0.68 |
| pH | --- | 6.5-8.5 |
| Moisture | % | 20 |
| Carrier | --- | Peat |

**Table S2** Phosphorus sources used in the experiment, with and without phosphate-solubilizing bacteria

| **Phosphorus source** | **Phosphorus dose** | **Phosphate-solubilizing bacteria (PSB)** | |
| --- | --- | --- | --- |
| Single superphosphate (SSP) | 45 kg ha^-1^ | No PSB |  |
| Rock phosphate (RP) | 90 kg ha^-1^ | With 5 kg ha^-1^ PSB |  |
